# Exposure to environmental radionuclides associates with tissue-specific impacts on telomerase expression and telomere length

**DOI:** 10.1038/s41598-018-37164-8

**Published:** 2019-01-29

**Authors:** Jenni Kesäniemi, Anton Lavrinienko, Eugene Tukalenko, Zbyszek Boratyński, Kati Kivisaari, Tapio Mappes, Gennadi Milinevsky, Anders Pape Møller, Timothy A. Mousseau, Phillip C. Watts

**Affiliations:** 1 Ecology and Genetics, University of Oulu, Oulu, 90014 Finland; 2 CIBIO/InBIO, Research Center in Biodiversity and Genetic Resources, University of Porto, PT-4485-661 Vairão, Portugal; 3 Department of Biological and Environmental Science, University of Jyväskylä, Jyväskylä, 40014 Finland; 4 Space Physics Laboratory, Taras Shevchenko National University of Kyiv, Kyiv, 01601 Ukraine; 5 Ecologie Systématique Evolution, Université Paris-Sud, CNRS, AgroParisTech, Université Paris-Saclay, F-91405 Orsay Cedex, France; 6 Department of Biological Sciences, University of South Carolina, Columbia, SC 29208 USA

## Abstract

Telomeres, the protective structures at the ends of chromosomes, can be shortened when individuals are exposed to stress. In some species, the enzyme telomerase is expressed in adult somatic tissues, and potentially protects or lengthens telomeres. Telomeres can be damaged by ionizing radiation and oxidative stress, although the effect of chronic exposure to elevated levels of radiation on telomere maintenance is unknown for natural populations. We quantified telomerase expression and telomere length (TL) in different tissues of the bank vole *Myodes glareolus*, collected from the Chernobyl Exclusion Zone, an environment heterogeneously contaminated with radionuclides, and from uncontaminated control sites elsewhere in Ukraine. Inhabiting the Chernobyl Exclusion Zone was associated with reduced TL in the liver and testis, and upregulation of telomerase in brain and liver. Thus upregulation of telomerase does not appear to associate with longer telomeres but may reflect protective functions other than telomere maintenance or an attempt to maintain shorter telomeres in a stressful environment. Tissue specific differences in the rate of telomere attrition and apparent radiosensitivity weaken the intra-individual correlation in telomere length among tissues in voles exposed to radionuclides. Our data show that ionizing radiation alters telomere homeostasis in wild animal populations in tissue specific ways.

## Introduction

Telomeres are nucleo-protein complexes that protect the ends of linear (*i*.*e*. eukaryotic) chromosomes, principally by preventing chromosome ends from fusing or being recognized as double strand DNA breaks^1^. Almost all vertebrate telomere sequences are composed of the motif TTAGGG that is repeated as a long, tandem array. Telomere length (TL) is reduced with cell division due to incomplete end replication^1^, with cell senescence triggered at a critically short TL^2^. As TL is widely correlated with longevity, health and fitness in humans and other animals, there is much interest in understanding the processes that impact TL dynamics. Stressors, such as psychological stress^3,4^, oxidative stress^5,6^ and environmental pollution^7^, correlate with accelerated rate of telomere shortening in humans and animals.

Telomere length homeostasis is more complex than the rate of shortening *per se*. A key mechanism by which telomeres may be repaired or extended is by the action of the enzyme telomerase^8^. Telomerase expression varies among tissues and among species^9,10^. Notably, small (~2 kg or less) rodents^9,11,12^ and some birds^13^ express telomerase in many somatic tissues as adults; conversely, many large long-lived mammals repress telomerase expression in somatic tissues as adults^14,15^, albeit with some exceptions (*e*.*g*. pig^16^). Moreover, the level of telomerase expression varies among tissues^9^. In adult mice (*Mus musculus* and *M*. *spretus)*, telomerase expression tends to be higher in tissues with longer telomeres (such as testis and liver), and weak or absent in tissues with shorter telomeres (such as spleen, kidney and brain)^17^. Similar pattern is seen in adult rats where telomerase is highly expressed in the liver, but has low expression in the brain tissue^18^. Stress also affects telomerase expression, but in an unpredictable way, with exposure to stress capable of stimulating and repressing telomerase activity^3,19–22^. Typically, inadequate expression of telomerase is associated with accelerated telomere shortening and aging^1,2^. However, an increase in telomerase expression is not always beneficial, for example with cancerous cells becoming immortalized by overexpression of telomerase^14^. Indeed, the level of telomerase expression for appropriate telomere maintenance likely interacts with species’ life-history, particularly whether animals tend to repress or express telomerase when mature. As the levels of expression of telomerase in wildlife exposed to stress and its impact on TL dynamics is unknown, quantification of TL dynamics and telomerase expression in different tissues and in animals under stress is needed.

Contaminated environments present complex stressors and health impacts on wildlife^23^. Numerous human actions have led to the accidental release of radionuclides into the environment, leaving many contaminated areas worldwide^24,25^. The wildlife inhabiting the area surrounding the former Nuclear Power Plant (NPP) at Chernobyl, Ukraine, provide the best-studied model of the biological impacts of exposure to environmental radionuclides^26^. On April 26, 1986, an explosion and subsequent fire at the Chernobyl NPP Unit 4 led to the release of more than 9 × 10^3^ PBq of radionuclides over much (>200,000 km^2^) of Europe and eastern Russia^26^. Subsequently, the Chernobyl Exclusion Zone (CEZ) was established at a 30 km radius around the site to limit human exposure to contamination. The organisms that inhabit the areas surrounding the Chernobyl NPP are exposed to elevated levels of radionuclides with long half-lives, notably strontium-90 (^90^Sr), cesium-137 (^137^Cs), and plutonium-239 (^239^Pu) that have half-lives of 29, 30 and 24,100 years respectively^26,27^. Diverse genomic impacts have been reported in wildlife inhabiting the areas surrounding Chernobyl^25^, including elevated levels of DNA damage^28^ and mutations^27^. However, these studies failed to identify specific genomic regions that are sensitive to damaging effects of inhabiting an area with elevated radioactivity.

We hypothesized that exposure to environmental radionuclides within the CEZ is likely to impact telomere homeostasis as telomeres present a specific target for damaging effects of ionizing radiation (IR) because G-rich regions of the genome are expected to be radiosensitive. Additionally, IR induced increase in cellular reactive oxygen species (ROS) and oxidative stress can disrupt telomere maintenance, as telomeres are sensitive to oxidative damage^5,19,29^. Indeed, both TL and telomerase expression are linked with radiosensitivity *in vitro*: cell lines with short telomeres show increased radiosensitivity when they lack a functioning telomerase^19^ (also *in vivo* in mice^30^). Other cell lines show a reduction in telomere length and an increase in telomerase expression after exposure to ionizing radiation^31^, and radiosensitive cells have accelerated telomere shortening and telomere dysfunction^32,33^. The extent to which wildlife experiencing a chronic, low dose exposure of ionizing radiation parallel these studies of cell lines exposed to acute, high doses of radiation (*e*.*g*. for clinical context) is unknown.

Radiosensitivity defines the relative susceptibility of cells, tissues, organs, organisms, or other substances to the injurious action of radiation. Radiosensitive cells are typically those with a high rate of division, high metabolic rate and/or are non-specialized^34^. Thus, TL in mice testis and skin is particularly sensitive to chronic oxidative stress due to the rapid rate of cell division in these cell types^35^. Liver, heart and brain tissues have high metabolic demands, but differ in their proliferative capabilities and production of ROS^36^. While tissues invariably cope with their intrinsic oxidative stress, the additional stress caused by exposure to radionuclides (i.e. inhabiting the CEZ) could pose a challenge for telomere maintenance.

In summary, different tissues possess an intrinsically different balance between telomere erosion and telomere repair and their radiosensitivity. The aim of this study was to quantify the effect that chronic exposure to ionizing radiation, associated with inhabiting the CEZ, has upon telomere homeostasis in wild populations by quantifying telomerase expression and TL in different tissues of the bank vole *Myodes glareolus*.

## Methods

### Model species

The bank vole *Myodes glareolus* is a muroid rodent that inhabits deciduous and coniferous woodland throughout much of northern Europe and Asia^37^. This species presents an ideal model to quantify biological effects of exposure to environmental radiation as it was one of the first mammals to recolonize areas contaminated by radionuclides following the Chernobyl accident^38^. Bank voles inhabiting the CEZ can experience considerable (*i*.*e*. >10 mGy/d, but average around 3.7 mGy/d) absorbed doses of radiation^38^. Evidence for genetic damage in bank voles inhabiting the CEZ is equivocal, with reports of increased levels of chromosomal aberrations contrasting with no evidence of increased micronuclei formation (reviewed by^25^) or accumulations of heteroplasmic mutations in mitochondrial DNA^39^. Nonetheless, bank voles inhabiting the CEZ upregulate some DNA repair enzymes^40^.

### Sample collection

**Figure 1:**
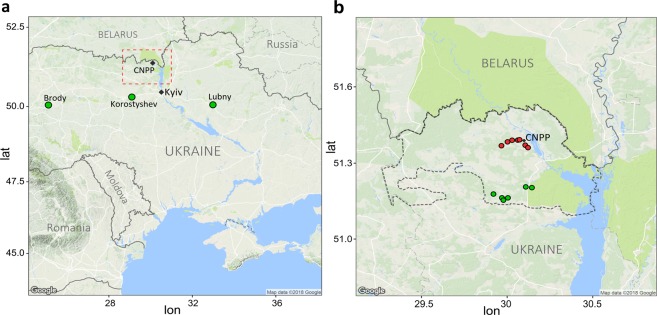
*Myodes glareolus* sampling sites. Location of uncontaminated control sampling sites (**a**) and the Chernobyl Exclusion Zone (CEZ, border marked with dotted line) in Ukraine (**b**). For the sampling locations within the CEZ (**b**), sites with ambient radiation > 1 µSv/h are marked red circles and sites with radiation level of <1 µSv/h are marked with green circles.

During 2015, bank voles were captured from 13 trapping locations within the CEZ and from control locations outside the CEZ: Brody, Lubny and Korostyshev (Fig. 1). The CEZ presents a mosaic of radionuclide contamination with areas of elevated levels of contamination and relatively uncontaminated areas across a broad landscape. Bank voles can move distance of 1 km during the breeding season^41^ and thus animals captured within the CEZ have the opportunity to be exposed to radionuclides prior to capture. In contrast, the control sites are located some 170 to 450 km from the CEZ and individuals from these locations would not have been exposed to environmental radionuclides. Levels of ambient ground-level soil radiation at trapping locations within the CEZ varied from 0.12 to 12.34 µSv/h (locations with elevated contamination: mean = 5.21 µSv/h, SD = 2.71 and clean areas with lower levels: mean = 0.19 µSv/h, SD = 0.11). The external radiation exposure of bank voles at Chernobyl strongly correlates (r = 0.86, Pearson) with the ambient background dose rates of the trapping sites (Lavrinienko *et al*. unpublished). The means for the uncontaminated control areas were 0.13 μSv/h, 0.14 μSv/h and 0.13 μSv/h for Brody, Lubny and Korostyshev, respectively. Thus, the level of soil background radiation was significantly different between the CEZ and control areas (t-test: t = 4.95, df = 58, P < 0.001).

All procedures complied with the legal requirements and international guidelines for the use of animals in research. All necessary permissions for the experiments were obtained from the Animal Experimentation Committee (permission no. ESAVI/7256/04.10.07/2014).

### Relative telomere length (RTL) and telomerase expression

We quantified relative TL in bank voles (N = 41) using the quantitative polymerase chain reaction (qPCR) method described by Cawthon, in which relative telomere length (RTL) is presented as the T/S ratio, where T is the amount of telomere in a sample and S reflects the amount of standard single copy gene (S). Additionally, the experimental samples are compared to a reference ‘golden standard’ DNA sample run on each qPCR plate^42^. TL was quantified in liver, brain, testis, ovary and heart tissue. Telomerase expression was quantified by qPCR in liver, brain, ovary and heart tissue. Telomerase expression is presented as relative quantities (fold change) to a reference cDNA sample that was present on all plates (see Supplementary Information for full details of both protocols).

### Statistical analyses

Differences in TL and telomerase expression between samples collected from within the CEZ and the control areas were analyzed using generalized linear models (GLM) in R v.3.4.3^43^, using sample area (CEZ, control) and sex as fixed factors. Intra-individual correlations for telomere length were examined for CEZ and control individuals separately, and pairwise comparison for all tissues were performed (Pearson’s correlation in R). REST 2.0.13 software^44^ was used for comparing general levels of telomerase expression among tissues.

## Results

### Telomere length

**Figure 2:**
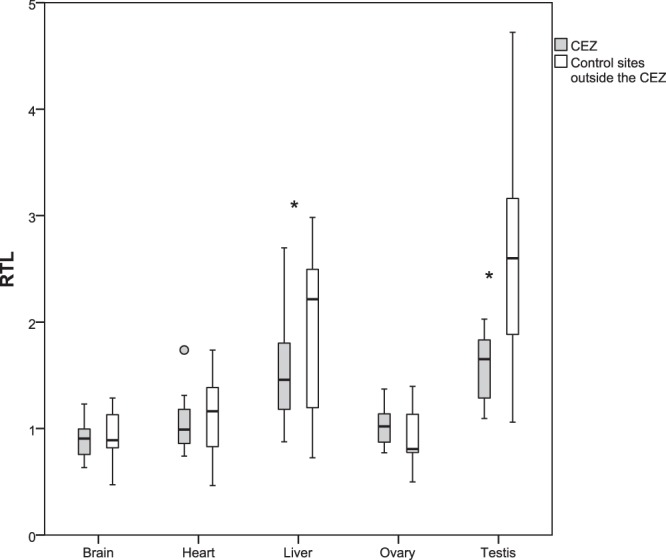
Telomere length. Relative telomere length (RTL) in the different tissues from bank voles from the Chernobyl Exclusion Zone (CEZ) and uncontaminated control sites. Box plots show medians, quartiles, 5- and 95-percentiles and extreme values. Statistically significant comparisons are marked with * (significant at the 0.05 level).

Relative telomere lengths were estimated in liver, brain, heart, testis and ovary tissue from bank voles within the CEZ and control animals from Ukraine which were not exposed to increased levels of ambient background radiation in their environment. Samples from the two groups within the CEZ were combined since animals from elevated radiation areas and relatively clean areas did not differ in TL. Animals from the CEZ had shorter telomeres compared to control animals in liver and testis. Brain, heart and ovary samples showed no differences between the two groups (Table 1, Fig. 2). Males had longer telomeres in all studied somatic tissues compared to females (Supplementary Information, Fig. S1). In general, telomeres were longer in liver and testis compared to brain, heart and ovary (Table 2, Fig. 2).

**Table 1:** Summary of GLM results for relative telomere length (RTL) and telomerase expression for bank voles within the CEZ and control sites outside the CEZ, and the effect of sex.

		Telomere length (RTL)				Telomerase expression			
		B	SE	*t*	*P*	B	SE	*t*	*P*
Brain	intercept	1.027	0.039	26.61	0.000	0.493	0.147	3.36	0.001
	Control	0.045	0.044	1.03	0.311	−0.481	0.167	−2.89	**0**.**006**
	Female	−0.281	0.044	−6.37	**0**.**000**	−0.025	0.167	−0.15	0.883
Heart	intercept	1.243	0.068	18.35	0.000	−1.952	0.251	−7.79	0.000
	Control	0.064	0.076	0.84	0.407	−0.382	0.358	−1.066	0.292
	Female	−0.375	0.076	−4.96	**0**.**000**	0.372	0.358	1.038	0.305
Liver	intercept	1.784	0.162	11.03	0.000	1.033	0.290	3.56	0.001
	Control	0.435	0.187	2.33	**0**.**026**	−0.878	0.320	−2.75	**0**.**008**
	Female	−0.508	0.187	−2.72	**0**.**009**	−0.311	0.320	−0.97	0.337
Ovary	intercept	1.026	0.104	9.86	0.000	0.105	0.118	0.896	0.380
	Control	−0.018	0.154	−0.12	0.908	−0.105	0.163	−0.646	0.525
Testis	intercept	1.594	0.258	6.17	**0**.**000**				
	Control	1.020	0.357	2.86	**0**.**010**				

Values in bold are statistically significant at the 0.05 level.

**Table 2:** Mean relative telomere length (RTL) and relative telomerase expression in bank vole tissues from animals from the Chernobyl Exclusion Zone (CEZ) and control sites outside the CEZ.

Tissue (N CEZ/control)	CEZ RTL (SD)	control RTL (SD)	combined
**RTL**			
Brain (20/21)	0.89 (0.17)	0.94 (0.22)	0.92 (0.20)
Heart (20/21)	1.06 (0.24)	1.13 (0.35)	1.10 (0.30)
Liver (19/21)	1.55 (0.46)	1.98 (0.76)	1.78 (0.66)
Ovary (12/10)	1.03 (0.18)	1.01 (0.49)	1.02 (0.35)
Testis (10/11)	1.59 (0.30)	2.61 (1.07)	2.13 (0.95)
**Tissue (N CEZ/control)**	**CEZ telomerase (SD)**	**control telomerase (SD)**	**combined**
**Telomerase**			
Brain (30/29)	1.69 (0.92)	1.17 (0.62)	1.44 (0.82)
Heart (25/24)	0.37 (0.28)	0.24 (0.17)	0.31 (0.25)
Liver (28/28)	0.80 (0.43)	0.54 (0.34)	0.67 (0.40)
Ovary (12/13)	1.25 (0.31)	1.16 (0.33)	1.20 (0.32)

N = sample size, SD = standard deviation.

### Telomerase

**Figure 3:**
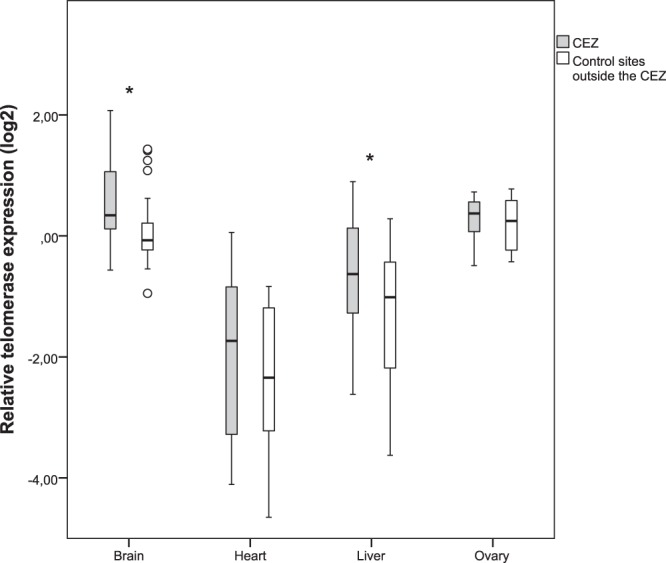
Telomerase expression. Relative telomerase expression in the different tissues from bank voles from the Chernobyl Exclusion Zone (CEZ) and uncontaminated control sites. Box plots show medians, quartiles, 5- and 95-percentiles and extreme values. Statistically significant comparisons are marked with * (significant at the 0.05 level).

Expression of telomerase was higher in the brain and ovary tissues compared to liver and heart (Fig. 3, Table 2). There was a 5.5 fold difference between the tissues with highest and lowest telomerase expression, brain and heart, respectively. Two tissues showed significant increase in telomerase expression in animals from the CEZ (Table 1). Telomerase was upregulated (1.4 fold change) in brain tissue of Chernobyl animals compared to individuals from control sites outside the CEZ, with no difference between males and females. No difference was seen between CEZ animals from elevated radiation or clean areas when three groups were analyzed. Increase in telomerase expression was also observed in liver tissue of Chernobyl voles compared to control individuals (1.7 fold change, no difference between sexes). In ovaries and heart tissue, there were no differences in telomerase expression between animals from Chernobyl and control areas (or between sexes for heart).

### Correlation in telomere length among tissues

In animals from uncontaminated control locations outside the CEZ, within individual correlations between telomere lengths in different tissues were positive and significant (pairwise correlations ranging from r = 0.50–0.82, excluding testis, Table 3), with the highest correlation seen between the post-mitotic heart and brain tissues (r = 0.82). The exception was testis, where patterns were weak and non-significant. However, in animals from Chernobyl, the intra-individual correlations in TL between tissues were weaker, and for example, correlations involving liver tissue were degraded (r = 0.14 in TL of liver and the somatic tissues brain and heart) (Table 3).

**Table 3:** Intra-individual Pearson correlations for telomere length in the different tissues.

	Brain	Heart	Liver	Ovary	Testis
Brain		0.40*	0.14	0.58*	−0.61*
Heart	0.82***		0.14	0.01	−0.21*
Liver	0.50**	0.55***		−0.13	0.61
Ovary	0.64**	0.80***	0.73**		—
Testis	0.27	0.14	0.40	—	

Values above the diagonal are within animals from the Chernobyl Exclusion Zone (CEZ) and values below the diagonal are from animals from uncontaminated control sites.

Significance level: ***< 0.001, **0.01, *0.05.

## Discussion

Genetic damage is a characteristic biomarker for organisms inhabiting areas contaminated by environmental radioactivity^25^, and previous studies have shown that mammalian cell lines with short telomeres and/or malfunctioning telomerase are radiosensitive^19,45^. In bank voles inhabiting the CEZ, exposure to environmental radioactivity is associated with upregulation of telomerase (principally in brain and liver) and a general reduction in telomere length (TL) (in liver and testis). Additionally, tissue-specific differences in the sensitivity to exposure to radionuclides disrupts the within-individual correlation in TL of different tissues. To the best of our knowledge this is the first evidence that exposure to environmental radionuclides impacts telomere homeostasis in wild animals.

Sex specific differences in TL are common in mammals, with adult females having longer telomeres than males due to a slower rate of telomere attrition throughout life^10,11^. Why female bank voles have shorter telomeres than male voles is unclear, but may reflect greater growth (cell division) in the larger females or higher costs of reproduction^46^. Additionally, the intracellular oxidative stress environment may differ among bank vole sexes; for example, female rats have greater protection against oxidative stress and longer telomeres^47^. Nonetheless, we found no interaction between sex and the effects of exposure to radionuclides on telomere homeostasis.

As exposure to stressful events impacts telomere dynamics^48,49^, and telomere length (TL) is equivalent among tissues of newborn animals^17,50^, any differences in TL among adult tissues reflects tissue-specific rates of erosion (*e*.*g*. cell division, oxidative stress) and repair (*e*.*g*. expression of telomerase). In bank vole, the apparently high radiosensitivity of the testes^34^ (*i*.*e*. the reduction in mean and variance in TL, Fig. 2) is consistent with the high rate of cell division in this tissue^35^. Nonetheless, telomeres of bank vole testes are longer than telomeres of somatic tissues, consistent with tissue specific variation in TL among mouse^17^, implying that it is critical to maintain long telomeres in testes. Maintenance of TL in bank vole testes is presumably achieved by expression of telomerase, as the testes are generally characterized by high telomerase expression, even in species that repress telomerase in somatic tissues as adults^9,14,51^. Our qPCRs confirmed expression of telomerase in bank vole testes in all sample locations, although we were unable to determine relative telomerase expression associated with exposure to radionuclides (see Supplementary Information). Repression of telomerase associated with inhabiting the CEZ is unlikely, given the general trend in the somatic bank vole tissues (Fig. 3), and that a lack of telomerase expression in testes has critical fitness implications (such as depletion of sperm, decrease in testis size and increased apoptosis associated with excessive telomere shortening)^52^. The shorter TL of testes in the contaminated areas is important as it implies that radionuclide contamination can impact the multigenerational dynamics of TL in wild mammal populations. In humans and other mammals there is evidence that TL is determined by the paternal TL at the age of conception, with offspring inheriting longer telomeres from older fathers with longer telomeres in the sperm cells^53^. Short telomeres in bank voles inhabiting the CEZ may reflect reproduction by younger males, for example due to accelerated mortality in the contaminated environment, and/or accelerated telomere erosion due to the stressful environment (and potentially inadequate repair by telomerase). Irrespective of the mechanism(s) associated with telomere attrition, young bank voles likely begin their lives with shorter telomeres than typical of bank voles born in uncontaminated environments.

While ovary tissue is considered to be sensitive to radiation induced damage^34^, neither TL nor telomerase expression in ovaries were notably impacted by exposure to radionuclides. Low telomerase expression is common in mature ovary tissue and oocytes in mammals (*e*.*g*. macaque *Macaca fascicularis* ovaries^54^, *Mus spretus* oocytes^55^) as in the bank vole. Some studies have implicated a role for maternal TL inheritance in mammals^56^. While the mode of telomere heritance is not known for bank voles, the short TL in ovaries is perhaps consistent with the putative role of paternal, not maternal, mode of TL inheritance.

The contrasting effects of exposure to radionuclides on TL of liver, brain and heart tissues appears consistent with their comparative radiosensitivity, with no differences in TL among the CEZ and control samples in brain and heart (Fig. 2), which are less sensitive to radiation damage than liver tissue^34^. However, the comparatively low TL in bank vole brain and heart could indicate that these tissues naturally experience telomere attrition during development, and the short telomeres in adults reduce statistical power to detect an effect of radiation. Samples of newborn and adolescent bank voles are required to determine whether exposure to radionuclides accelerate telomere erosion during development. Given the short telomeres in bank vole brain and heart, it is notable that telomerase expression is low or absent in mouse and rat brain and heart tissues^17,18,35^, and similar pattern of expression occurs in other rodents and birds as well^9,13^. However, while telomerase expression was also low in bank vole heart, there was relatively high expression of telomerase in brain tissue, and upregulation of this enzyme in voles inhabiting the CEZ. This potentially indicates an attempt to prevent telomeres of the brains from becoming critically short in adults. It is possible that during adulthood, bank vole brain tissue is somewhat resistant to telomere attrition, as brains do not show age-related telomere shortening in rats^10^ and there is low mitochondrial ROS production in rodent brains^36^. Alternatively, telomerase may have wider functions than telomere maintenance, such as protection of cellular and mitochondrial function during oxidative stress^57^, inhibition of apoptosis^58^ and/or activation of DNA repair pathways^59^. Interestingly, small brain size is a characteristic malformation in birds affected by radioactive fallout from the CEZ^60^ and in adult bank voles inhabiting contaminated areas of the CEZ (Kivisaari *et al*. unpublished). Small brain size can imply fewer cell divisions during development, and this may be a further mechanism that limits the extent that exposure to radionuclides erodes TL. Whether small brain size could represent an ‘adaptation’ by bank voles inhabiting the CEZ, is an intriguing topic for further study.

Many (but not all) small rodents express telomerase in their livers as adults^9,18^ and have inherently long (compared to brain and kidney in mouse^17^) telomeres as adults. Long telomeres (relative to heart and brain) and expression of telomerase indicate a need to maintain telomere length in adult bank vole liver. Nonetheless, exposure to environmental stress impacts TL dynamics in the liver tissue, as inhabiting CEZ was associated with a reduction in TL and upregulation of telomerase. To highlight the complex relationship between telomerase expression and TL, the increased expression of telomerase in the liver appears insufficient to elongate/maintain TL in bank voles inhabiting the CEZ, but as noted above (for brain tissue) telomerase may have pleiotropic roles associated with the molecular stress response beyond safeguarding telomeres. Interestingly, expression of *Mre11*, which is involved with telomere maintenance, is upregulated in CEZ bank vole livers^40^. In any case, we find the liver tissue of adult bank voles exhibits some radiosensitivity as expected^34^.

In addition to the impact of telomerase, telomere length homeostasis can be regulated via multiple other mechanisms, for example recombination-mediated alternative lengthening of telomeres (ALT^61^) or stochastic events such as telomere rapid deletions (TDR^62^) can lengthen or shorten telomeres^63,64^. However the relevance of these is unclear especially in species and tissues where telomerase in continuously expressed, such as in the bank vole.

The similar patterns of telomere dynamics in animals inhabiting the CEZ, irrespective of the background level of contamination, is interesting in light of a recent debate about the possible role of historic dose influencing contemporary biological response^65^. As bank voles can disperse among the mosaic of radionuclide contamination within the CEZ, animals inhabiting apparently uncontaminated areas may have experienced radionuclide contamination at some point in their life. This prior experience of radionuclide exposure may trigger a persistent biological response, and epigenetic effects may even persist across generations^66^. Alternatively, changes in telomere homeostasis might reflect persistent adaptation(s) to radionuclide contamination that was driven by selection in the population of bank voles that recolonized the CEZ soon after the accident.

Despite the intrinsic tissue-specific variation in the rate of telomere attrition during an individual’s life, a positive correlation in telomere lengths among tissues within individuals is typical in humans^54,67^ and other animals^54,68,69^. This intra-individual correlation in TL exists in natural populations of bank voles, except for comparisons involving the testis where telomere lengths are long and variable. A crucial outcome of our analysis is that exposure to environmental radioactivity (and potentially other stressors) weakens or removes the intra-individual correlation in TL, presumably due to tissue-specific differences in radiosensitivity that impact telomere homeostasis (*i*.*e*. the rate of telomere attrition and effects of telomerase) in some tissues more than others.

Exposure to stress can induce telomerase expression in animals^19,21,22^. Upregulation of telomerase and short TL characterizes bank voles exposed to environmental radionuclides in the CEZ. The impact on telomere dynamics in humans is unclear, as contrasting results of either slight lengthening^70^, or shortening^71^ of leukocyte telomeres have been reported in studies of the late effects of exposure to radioactive fallout in Chernobyl clean-up workers. Humans can increase telomerase expression in response to acute stress^20^, but telomerase expression associated with exposure to environmental radiation in humans is unknown. Of course, it is hard to compare these human data with our study as bank voles are continually exposed to radionuclides in soil and internally via their diet; moreover, small and large animals have different telomere homeostasis, notably in the pattern of telomerase expression in somatic tissues of adults^9,12^. However, our data on telomerase are comparable with studies on cell lines; upregulation of telomerase is a common response by cell lines to acute exposure to ionizing radiation (>1 Gy)^19,31^. Given the tissue specific differences in telomerase expression and telomere dynamics in bank voles, further work on other mechanisms possibly affecting telomere dynamics in response to stress is required. For example, methylation levels at subtelomeric regions can impact telomere dynamics by regulating the frequency of telomeric recombination events or the expression of genes involved in telomere maintenance^72^, and may be relevant for telomere maintenance in species inhabiting the CEZ, as radiation exposure has been shown to alter genome wide methylation levels in *Pinus silvestris* from the CEZ^73^.

Currently, telomerase expression is a neglected aspect of our understanding of telomere dynamics in wild animal populations (but see^13^), however it is important to study both TL and telomerase expression to understand telomere homeostasis, especially in species expressing telomerase in somatic tissues as adults. Thus, while TL and rate of telomere loss are widely associated with lifespan^74^, expression of telomerase is another mechanism associated with (cellular) longevity^75,76^. We also caution against an interpretation that the generally short telomere length associated with the CEZ is evidence that telomerase does not maintain telomeres or that telomerase is directed towards other molecular functions. As telomeres are sensitive to damage by factors that increase oxidative stress (such as radiation), it is not clear whether long telomeres *per se* would be better adapted to a stressful environment. For example, murine cell lines or mice with short telomeres and which lack telomerase expression are the most sensitive to acute exposure to ionizing radiation^19,30,77^, indicating that telomerase could protect short telomeres against radiation-induced damage^78^. Longer telomeres present a greater target (physical length) for damage by radiation, and excessively long telomeres can confer genomic instability^79^. Therefore, a combination of short telomeres that are better protected/repaired (*i*.*e*. via telomerase expression), rather than attempting to maintain long telomeres, may be a more effective method of maintaining cell function and genomic stability in areas contaminated by radionuclides. Bearing in mind the potential effects of short TL in testis, future studies need to quantify whether short telomeres are adaptive (*i*.*e*. easier to maintain and repair) within the CEZ, or whether short telomeres are a passive reflection of life within a stressful environment.

## Supplementary information

Supplementary Information

## Data Availability

The datasets analysed during the current study are available from the corresponding author on reasonable request.
