# Supplementary material for: Exposure to environmental radionuclides associates with tissue-specific impacts on telomerase expression and telomere length
Source: Sci Rep. 2019 Jan 29;9:850. doi: 10.1038/s41598-018-37164-8 (PMC6351625; doi:10.1038/s41598-018-37164-8)
Supplement: Supplementary file 1 — Supplementary Information [file 41598_2018_37164_MOESM1_ESM.pdf]

Supplementary Information

Exposure to environmental radionuclides associates with tissue-specific impacts on telomerase expression and telomere length

Jenni Kesäniemi, Anton Lavrinienko, Eugene Tukalenko, Zbyszek Boratyński, Kati Kivisaari, Tapio Mappes, Gennadi Milinevsky, Anders Pape Møller, Timothy A. Mousseau, Phillip C. Watts

Materials and methods

Sample collection

Bank voles were trapped using Ugglan Special live traps (Grahnab, Sweden), with sunflower seeds and potato as bait. At each sampling area, 10 traps were placed in line, with each trap separated by about 10 m. Levels of ambient environmental radiation were measured at ground level at each trapping site using a hand-held GM dosimeter (Inspector, International Medcom INC, Sebastopol, CA, USA) calibrated to measure Sieverts (Sv/h).

All animals were euthanized by cervical dislocation and tissue samples collected and transferred immediately to Allprotect Tissue Reagent (Qiagen) or RNAlater (Qiagen), to provide RNA to quantify telomerase expression, or transferred to EtOH to provide DNA to quantify relative telomere length (RTL). Samples were stored at -80°C (AllProtect, RNAlater) or -20°C (EtOH) prior to further processing. All procedures complied with the legal requirements and international guidelines for the use of animals in research. All necessary permissions for the experiments were obtained from the Animal Experimentation Committee (permission no. ESAVI/7256/04.10.07/2014).

Relative telomere length (RTL)

To estimate variation in telomere length among samples, DNA was extracted from liver, brain, testes, ovary and heart tissue using a DNeasy kit (Qiagen). DNA concentrations were measured using a NanoDrop spectrophotometer (ThermoFisher) and normalised to 5 ng/μl. Relative telomere length (RTL) was quantified using a combination of two quantitative polymerase chain reactions (qPCR) as described by Cawthon[1]. The first qPCRs amplified telomeric regions using vertebrate telomere primers designed by Cawthon[1]; the second qPCRs amplified part (87 bp) of the single copy gene (*36B4* [=*Rplp0*] that encodes the ribosomal phosphoprotein P0) that was used as a non-variable standard. Primers to amplify *36B4*, designed against a draft bank vole genome (Genbank accession no. GCA_001305785), were 5’-GTC CCG TGT GAA GTC ACT GT-3’ and 5’-AGC GGT GTT GTC TAA AGC CT-3’.

The qPCRs were completed on a CFX96 (BioRad) machine, with telomere and *36B4* reactions performed on different plates, but with an identical sample layout for all samples. Each qPCR

contained ~20 ng DNA in a 15 µl final reaction volume that contained 0.3 µM each primer and 1X of iQ SYRB Green supermix (Bio-Rad); the $MgCl_2$ concentration was raised by 1.5 mM in the *36B4* qPCRs. Thermal cycling conditions were: 10 min at 95°C, followed by 40 cycles of 95°C for 15 sec and 55°C for 1 min followed by melt curve measurement from 65°C to 95°C (at a 0.5°C/5sec increase). All qPCRs were completed in triplicate to provide a mean quantification cycle (Cq) value per sample; mean Cq values were calculated from two qPCRs if the standard deviation (SD) among triplicates was high (>0.2), or sample was rerun if the SD among duplicate qPCRs remained high. In addition to the samples, every qPCR plate contained (1) the same 'golden standard' sample (DNA from bank vole muscle tissue normalised to 5 ng/µl), (2) no-template controls to confirm that reagents were not contaminated, and (3) a serial dilution of DNA that was used to calculate qPCR efficiency (E) for the plate and to ensure that the reactions occurred in the linear range: serial DNA dilutions were an 1:8 dilution (starting from 40 ng/µl). Mean efficiencies of qPCRs were 1.91 (n=8) and 2.01 (n=8) for the telomere and *36B4* reactions respectively; the mean inter-plate coefficient of variation of the telomere and 36B4 qPCR runs was 1.31% and 1.29% respectively, as calculated from variation in the golden standard sample. Reproducibility of the qPCRs was quantified by making two separate estimates of RTL for 40 bank vole DNA samples on different qPCR plates; Cq values for the telomere (R=0.95, P<2.2*10^{-16}) and *36B4* (R^2=0.98, P<2.2*10^{-16}) qPCRs were positively correlated, and thus the estimates of relative telomere length (see below for calculations) were also positively correlated (R=0.87, P=4.0*10^{-13}) among different runs. Relative telomere length (RTL) was calculated using Pfaffl's[2] formula to account for variation in qPCR efficiencies; briefly, the amount of telomere (T) in a sample is calculated as T=E^(Cq_{GS}–Cq_{sample}), where E is the qPCR efficiency, and Cq_{GS} and Cq_{sample} are the average Cq values in the golden standard and the sample, respectively; similarly, the amount of standard (S) gene in a sample is calculated as S=E^(Cq_{GS}–Cq_{sample}). The RTL is the ratio T/S.

Telomerase expression

RNA extraction for all tissue samples was done using RNeasy Mini Kit (Qiagen) with sample homogenization using TissueLyser II (Qiagen) and an on-column DNase digestion step. 400 ng of total RNA per sample was used for reverse transcription using iScript cDNA synthesis kit (Bio-Rad). Primers to amplify (1) telomerase reverse transcriptase (*Tert*) and (2) the reference gene Retention in endoplasmic reticulum sorting receptor 1 (*Rer1*)[3] were designed against sequences from draft bank vole genome data. The primers spanned exon-exon junctions to prevent

amplification of any residual genomic DNA, using information on exon-intron boundaries in *M. musculus* (from www.ensembl.org). Telomerase (*Tert*) primers (5’-CTA ACC TCC TGG GGG CTA CT-3’ and 5’-CCG CCA CAT CTG CCT TAA CA-3’) amplified a 126 bp product, and *Rer1* primers (5’-GGC CGA TCC TGG TGA TGT AC-3’ and 5’-CCA CGT CCT CCT TCC CTT TG-3’) produced a 132 bp amplicon. *Rer1* was a suitable reference gene as it showed stable expression within and among tissues (*n*=24 samples analysed for each tissue, brain: Cq=24.37, SD=0.22; liver: Cq=24.14, SD=0.43; ovary: Cq=24.26, SD=0.24). We could not calculate relative telomerase expression in testes, as it was impossible to identify a suitable reference gene for this tissue, with variation of *Rer1* Cq values being very high (mean Cq=26.11, SD=2.2, N=16), and similar for 3 other tested reference genes (*Actb, Ppia, Hprt*).

Quantitative PCR reactions (qPCRs) for gene expression were completed on a LightCycler480 Real-Time PCR System (Roche) in a 16 μl final reaction volume that contained 2 ng cDNA template, 0.5 μM of both primers and 1X of SYBR Green I Master Mix (Roche). Thermal cycling conditions were: 95°C for 5 min followed by 35 cycles of 95°C for 10 sec, 60°C for 15 sec and 72°C for 10 sec. Three technical replicates were run for every sample and qPCRs were repeated when the standard deviation (SD) among replicates was >0.2. Primer efficiencies, calculated from a 5-fold serial dilution of a mixture of cDNA from different tissues, were 2.04 and 1.98 for *Tert* and *Rer1,* respectively.

Telomerase expression data (Cp values) were analysed using GenEx v.6.1, where Cp values were efficiency corrected, interplate calibration was applied, technical repeats were averaged and samples were normalized with the reference gene. Following this, relative quantities (fold change) were calculated by setting a cDNA mix sample (‘golden standard’) run on all plates as the reference sample. Telomerase expression data were $\log_2$ transformed to achieve normal distributions, and exported for statistical analyses.

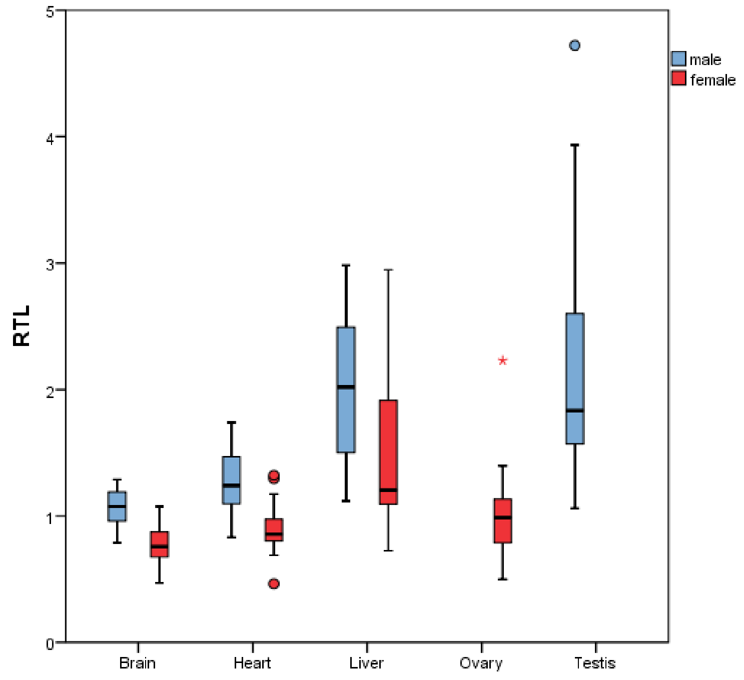

Fig S1. Sex specific differences in relative telomere length (RTL) in different tissues of male and female bank voles. Box plots show medians, quartiles, 5- and 95-percentiles and extreme values.

References

1. Cawthon, R. M. Telomere measurement by quantitative PCR. *Nucleic Acids Res.* **30,** 47e–47 (2002).
2. Pfaffl, M. W. A new mathematical model for relative quantification in real-time RT-PCR. *Nucleic Acids Res.* **29,** 45e–45 (2001).
3. Jernfors, T. *et al.* Transcriptional Upregulation of DNA Damage Response Genes in Bank Voles (Myodes glareolus) Inhabiting the Chernobyl Exclusion Zone. *Front. Environ. Sci.* **5,** (2018).
